# Supplementary material for: Workload Is Associated with Anxiety and Insomnia Symptoms in an Italian Nationally Representative Sample of Public Health Medical Residents: The PHRASI Cross-Sectional Study
Source: Healthcare (Basel). 2024 Nov 17;12(22):2299. doi: 10.3390/healthcare12222299 (PMC11593674; doi:10.3390/healthcare12222299)

Supplementary materials

Table S1 Linear regression models for anxiety symptoms (GAD-2)

	Model 1[a]				Model 2[b]				Model 3[c]			
**Variabile**	**Beta**	**2.5%**	**97.5%**	**p-value**	**Beta**	**2.5%**	**97.5%**	**p-value**	**Beta**	**2.5%**	**97.5%**	**p-value**
						Objective workload						
Additional employment (Yes)	0.097	-0.254	0.450	0.585	0.066	-0.285	0.417	0.712	0.055	-0.293	0.403	0.757
Working hours of additional contract valid for residency completion (Yes)	-0.740	-1.330	-0.150	**0.014**	-0.743	-1.334	-0.151	**0.014**	-0.791	-1.386	-0.196	**0.009**
Weekly working hours	-0.012	-0.031	0.008	0.25	-0.012	-0.032	0.008	0.225	-0.014	-0.033	0.006	0.175
Simultaneous traineeships (Yes)	0.483	0.019	0.947	**0.041**	0.498	0.038	0.959	**0.034**	0.443	-0.016	0.901	0.059
Work-life interference (Frequent)	1.035	0.626	1.445	**<0.001**	1.038	0.630	1.445	**<0.001**	0.990	0.583	1.397	**<0.001**
						Perceived workload						
High workload perceived (Yes)	0.806	0.335	1.277	**<0.001**	0.790	0.322	1.258	**0.001**	0.767	0.301	1.234	**0.001**
Work-SoC Manageability	-0.415	-0.536	-0.295	**<0.001**	-0.405	-0.527	-0.283	**<0.001**	-0.401	-0.520	-0.281	**<0.001**

Statistically significant p-values are in bold. [a] Adjusted for sex and age; [b] Adjusted for sex, age and economic status; [c] Adjusted for sex, age, physical activity and alcohol abuse

Table S2 Relation between sample characteristics and level of insomnia (ISI)

**Characteristic**	**No insomnia (ISI <8), N = 1921**	**Subclinical insomnia (8≤ ISI <15), N = 1311**	**Moderate insomnia (15≤ ISI <22), N = 371**	**Severe insomnia (ISI ≥22), N = 51**	**p-value[2]**
		Objective workload			
Additional employment					0.4
No	19 (51%)	4 (80%)	84 (64%)	126 (66%)	
Yes	18 (49%)	1 (20%)	47 (36%)	66 (34%)	

Working hours of additional contract valid for residency completion					0.9
No	11 (69%)	1 (100%)	33 (70%)	40 (65%)	
Yes	5 (31%)	0 (0%)	14 (30%)	22 (35%)	
Weekly working hours	38 (38, 47)	38 (38, 38)	38 (38, 38)	38 (38, 38)	0.5
Simultaneous traineeships					**0.002**
No	28 (76%)	1 (20%)	111 (85%)	168 (88%)	
Yes	9 (24%)	4 (80%)	20 (15%)	24 (13%)	
Work-life interference					**0.006**
Infrequent	22 (59%)	3 (60%)	107 (82%)	161 (84%)	
Frequent	15 (41%)	2 (40%)	24 (18%)	31 (16%)	
Perceived workload					
High workload perceived					**0.042**
No	26 (70%)	5 (100%)	113 (86%)	170 (89%)	
Yes	11 (30%)	0 (0%)	18 (14%)	22 (11%)	
Work-SoC Manageability	3.50 (2.50, 4.00)	3.50 (3.00, 3.50)	4.00 (3.25, 5.00)	4.50 (3.50, 5.00)	**<0.001**

Statistically significant p-values are in bold. [1] Median (IQR); n (%);[2] Kruskal-Wallis rank sum test; Fisher's Exact Test for Count Data with simulated p-value (based on 10000 replicates)

Table S3 Linear regression models for insomnia symptoms (ISI)

	**Model 1[a]**				**Model 2[b]**				**Model 3[c]**			
**Variabile**	**Beta**	**2.5%**	**97.5%**	**p-value**	**Beta**	**2.5%**	**97.5%**	**p-value**	**Beta**	**2.5%**	**97.5%**	**p-value**
Objective workload												
Additional employment (Yes)	0.564	-0.582	1.710	0.334	0.538	-0.608	1.685	0.357	0.573	-0.574	1.719	0.328
Working hours of additional contract valid for residency completion (Yes)	-0.89	-2.914	1.134	0.386	0.887	-1.124	2.898	0.387	1.031	-0.998	3.060	0.319
Weekly working hours	0.047	-0.017	0.111	0.147	0.047	-0.017	0.111	0.149	0.048	-0.016	0.112	0.138
Simultaneous traineeships (Yes)	2.459	0.973	3.946	**0.001**	2.474	0.992	3.957	**0.001**	2.475	0.988	3.962	**0.001**
Work-life interference (Frequent)	2.204	0.844	3.563	**0.001**	2.210	0.849	3.570	**0.001**	2.223	0.857	3.589	**0.001**
Perceived workload												
High workload perceived (Yes)	1.64	0.069	3.210	**0.041**	1.627	0.060	3.195	**0.042**	1.614	0.042	3.185	**0.044**
Work-SoC Manageability	-0.859	-1.272	-0.447	**<0.001**	-0.860	-1.277	-0.443	**<0.001**	-0.870	-1.282	-0.457	**<0.001**

Statistically significant p-values are in bold. [a] Adjusted for sex and age; [b] Adjusted for sex, age and economic status; [c] Adjusted for sex, age, physical activity and alcohol abuse

Table S4 Relation between workload characteristics and anxiety symptoms and moderation by sex

			GAD-2 score ≥ 3	
**Characteristic**	**Moderator**	**aOR[1]**	**95%CI**	**p-value**
	Objective workload			
Additional employment (Ref. No)	Predictor	1.05	0.59; 1.87	0.87
	Interaction	1.24	0.51; 3.01	0.64
Working hours of additional contract valid for residency completion (Ref. No)	Predictor	0.42	0.15; 1.16	0.09
	Interaction	1.34	0.29-6.27	0.71
Weekly working hours	Predictor	0.98	0.95 -1.02	0.31
	Interaction	1.01	0.96 -1.06	0.79
Simultaneous traineeships (Ref. No)	Predictor	1.73	0.81-3.72	0.16
	Interaction	0.53	0.16-1.75	0.3
Work-life interference (Ref. Infrequent)	Predictor	3.39	1.72-6.68	**<0.01**
	Interaction	0.69	0.24-2.02	0.5
	Perceived workload			
High workload perceived (Ref. No)	Predictor	2.53	1.22; 5.23	**0.01**
	Interaction	1.35	0.39; 4.73	0.64
Work-SoC Manageability	Predictor	0.61	0.48-0.76	**<0.01**
	Interaction	1.15	0.81; 1.63	0.43

Statistically significant p-values are in bold. [1]Adjusted for age

Table S5 Relation between workload characteristics and insomnia symptoms and moderation by sex

			ISI score ≥ 15	
**Characteristic**	**Moderator**	**aOR[1]**	**95% CI**	**p-value**
	Objective workload			
Additional employment (Ref. No)	Predictor	0.81	0.28; 2.39	0.70
	Interaction	2.98	0.74; 12.0	0.13
Working hours of additional contract valid for residency completion (Ref. No)	Predictor	2.66	0.28; 25.39	0.4
	Interaction	0.25	0.02; 3.52	0.31
Weekly working hours	Predictor	1.02	0.96 ; 1.07	0.58
	Interaction	1.00	0.93 ; 1.08	0.95
Simultaneous traineeships (Ref. No)	Predictor	4.92	1.72; 14.04	**<0.01**
	Interaction	0.34	0.08; 1.51	0.16
Work-life interference (Ref. Infrequent)	Predictor	4.35	1.60; 11.80	**<0.01**
	Interaction	0.62	0.15; 2.48	0.50
	Perceived workload			
High workload perceived (Ref. No)	Predictor	2.43	0.80; 7.44	0.12
	Interaction	1.24	0.25; 6.08	0.79

Work-SoC Manageability	Predictor	0.63	0.45; 0.89	**<0.01**
	Interaction	1.05	0.64; 1.70	0.86

Statistically significant p-values are in bold. [1]Adjusted for age

Figure S1 Combined effects of each predictor and sex on anxiety symptoms

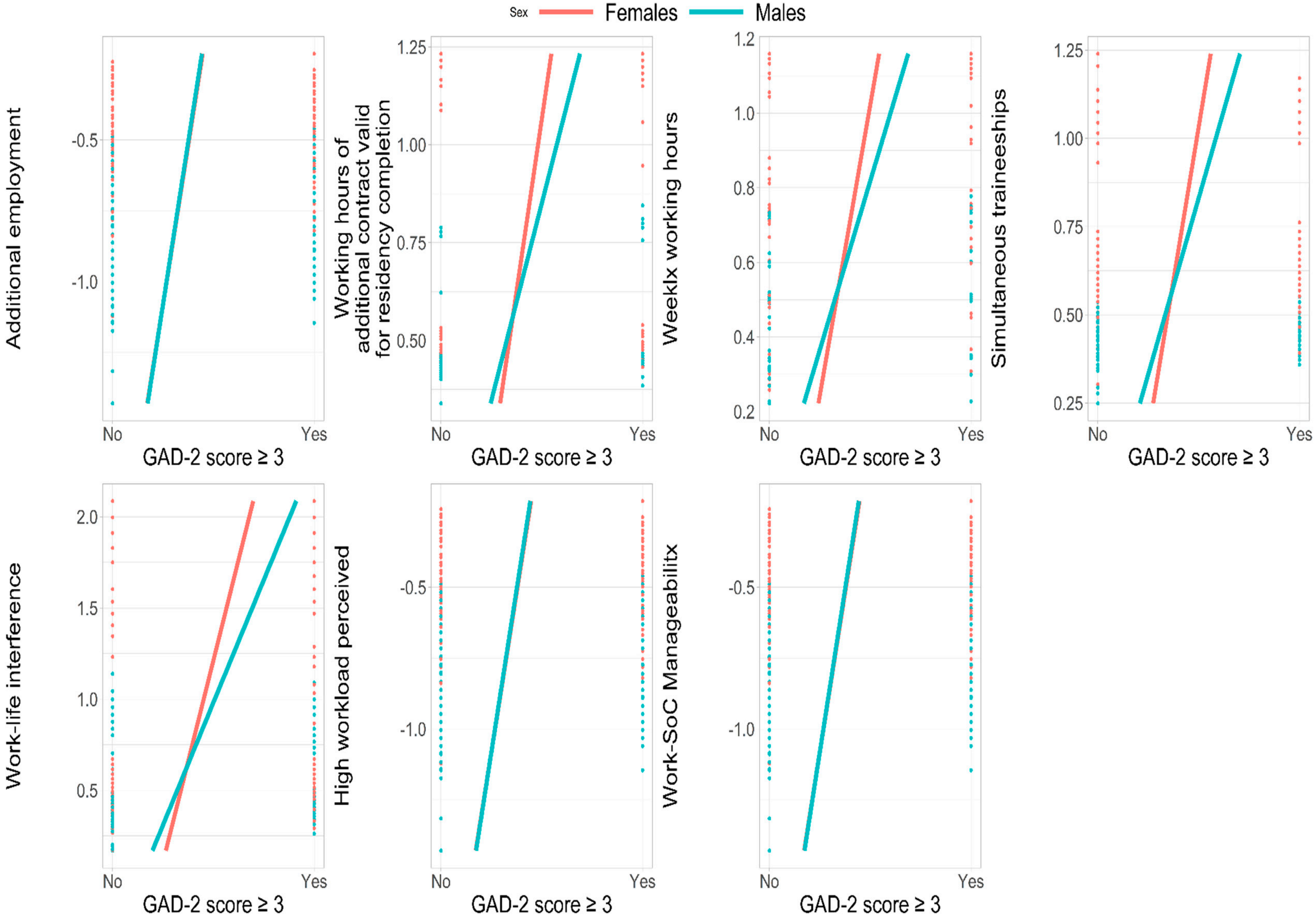

Figure S2 Combined effects of each predictor and sex on insomnia symptoms

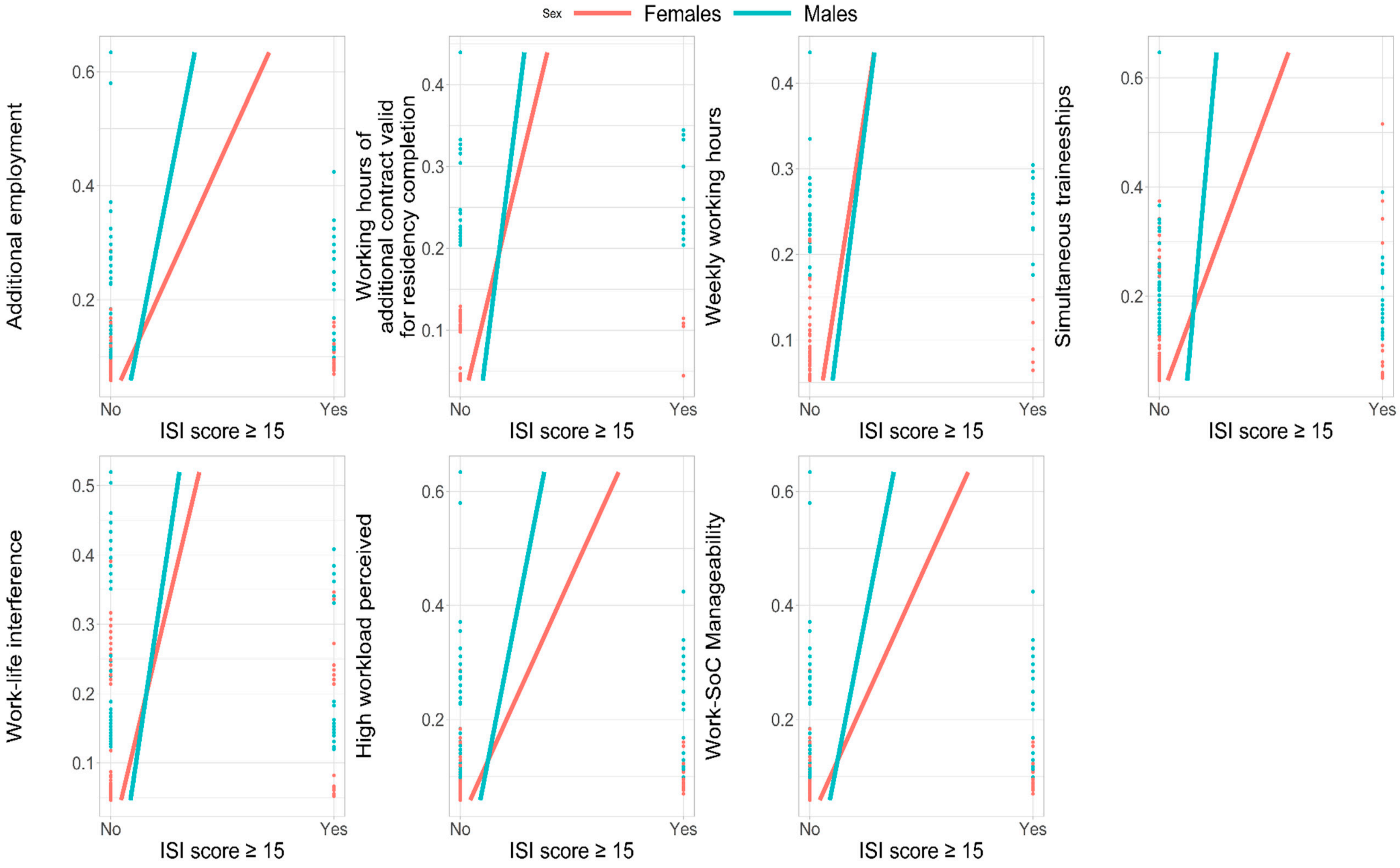

Supplement: Supplementary file 1 [file healthcare-12-02299-s001.zip › healthcare-3218223-supplementary.pdf]
